# Oligomeric and phosphorylated alpha-synuclein as potential CSF biomarkers for Parkinson’s disease

**DOI:** 10.1186/s13024-016-0072-9

**Published:** 2016-01-19

**Authors:** Nour K. Majbour, Nishant N. Vaikath, Karin D. van Dijk, Mustafa T. Ardah, Shiji Varghese, Louise B. Vesterager, Liliana P. Montezinho, Stephen Poole, Bared Safieh-Garabedian, Takahiko Tokuda, Charlotte E. Teunissen, Henk W. Berendse, Wilma D. J. van de Berg, Omar M. A. El-Agnaf

**Affiliations:** Department of Biochemistry, College of Medicine and Health Sciences, United Arab Emirates University, Al Ain, United Arab Emirates; Department of Anatomy and Neurosciences, Neuroscience Campus Amsterdam, VU University Medical Centre, Amsterdam, The Netherlands; Neural Plasticity and Repair Unit, Department of Experimental Medical Sciences, Wallenberg Neuroscience Center, BMC A10, Lund University, Lund, Sweden; Department of Neurodegeneration, H. Lundbeck A/S, 2500, Valby, Denmark; Biotherapeutics Group, National Institute for Biological Standards and Control, Potters Bar, Herts UK; College of Medicine, Qatar University, Doha, Qatar; Department of Neurology, Research Institute for Geriatrics, Kyoto Prefectural University of Medicine, Kyoto, 602-0841 Japan; Neurochemistry Laboratory and Biobank, Department of Clinical Chemistry, Neuroscience Campus Amsterdam, VU University Medical Centre, Amsterdam, The Netherlands; Department of Neurology, Neuroscience Campus Amsterdam, VU University Medical Centre, Amsterdam, The Netherlands; Neurological Disorders Center, Qatar Biomedical Research Institute, and College of Science and Engineering, Hamad Bin Khalifa University (HBKU), Education City, Qatar Foundation, P.O. Box 5825, Doha, Qatar

**Keywords:** Parkinson’s disease, Alpha synuclein, Cerebrospinal fluid biomarkers, Amyloid oligomers, Biomarkers

## Abstract

**Background:**

Despite decades of intensive research, to date, there is no accepted diagnosis for Parkinson’s disease (PD) based on biochemical analysis of blood or CSF. However, neurodegeneration in the brains of PD patients begins several years before the manifestation of the clinical symptoms, pointing to serious flaw/limitations in this approach.

**Results:**

To explore the potential use of alpha-synuclein (α-syn) species as candidate biomarkers for PD, we generated specific antibodies directed against wide array of α-syn species, namely total-, oligomeric- and phosphorylated-Ser129-α-syn (t-, o- and p-S129-α-syn). Next we sought to employ our antibodies to develop highly specific ELISA assays to quantify α-syn species in biological samples. Finally we verified the usefulness of our assays in CSF samples from 46 PD patients and 48 age-matched healthy controls. We also assessed the discriminating power of combining multiple CSF α-syn species with classical Alzheimer’s disease biomarkers. The combination of CSF o-/t-α-syn, p-S129-α-syn and p-tau provided the best fitting predictive model for discriminating PD patients from controls. Moreover, CSF o-α-syn levels correlated significantly with the severity of PD motor symptoms (*r* = -0.37).

**Conclusion:**

Our new ELISA assays can serve as research tools to address the unmet need for reliable CSF biomarkers for PD and related disorders.

**Electronic supplementary material:**

The online version of this article (doi:10.1186/s13024-016-0072-9) contains supplementary material, which is available to authorized users.

## Background

Parkinson’s disease (PD) is the second most common neurodegenerative disorder after Alzheimer’s disease. PD is characterized by a large time-gap between the beginning of the neurodegenerative processes and the onset of clinical manifestations [1]. However, its diagnosis is primarily based on motor-related clinical criteria. The natural history of the disease includes an initial asymptomatic stage followed by a long pre-motor phase; finally, when the classical motor symptoms appear, a minimum of 30 % of nigral dopaminergic neurons have already degenerated [2–5]. Therefore, there is an urgent need for the discovery of reliable biomarkers for PD diagnosis and prognosis. Toward this aim, recent intensive investigations have attempted to identify biomarker (s) to enable an early diagnosis of PD, preferably during the pre-motor phase. And since molecular changes in the brain can be reflected in the CSF proteome, this makes CSF an ideal source of reliable biomarkers [6]. Aggregated and hyper-phosphorylated forms of alpha-synuclein (α-syn) protein at S129 are the major components of Lewy bodies (LBs) and Lewy neurites and constitute the hallmark pathology in the brains of patients with PD and dementia with Lewy bodies [7]. Although the accumulation of α-syn is central to the development of synucleinopathies, the precise pathophysiological mechanism remains uncertain [8]. Of the different species of α-syn, intermediate oligomers/protofibrils are thought to be the major neurotoxic species [9, 10]. Therefore, developing therapeutic and diagnostic strategies that target these species is of paramount importance. Several retrospective studies have explored CSF α-syn as a putative biomarker for PD. A clear trend of lower CSF t-α-syn levels or marginally higher p-S129-α-syn/t-α-syn ratio in PD have been reported, although a large overlap between PD and control groups was noted [11–17]. Clearly, from previous studies it is evident that t-α-syn levels alone cannot discriminate individual patients with synucleinopathies from healthy individuals or those with other neurodegenerative diseases. Soluble α-syn oligomers (o-α-syn) have been increasingly linked to synaptic and neuronal degeneration [18] . The exact mechanism by which α-syn oligomers cause neuronal cell death is not fully elucidated. It has been shown that soluble o-α-syn is elevated in brain homogenates from PD and DLB cases compared with controls [19, 20]. Interestingly, we have previously reported that CSF o-α-syn levels were significantly higher in PD patients compared with other neurological disorders [14, 16]. CSF o-α-syn levels and o-α-syn/t-α-syn ratios were found to be substantially higher in patients with PD, including those with mild and early-stage disease, compared with controls [14, 16, 21–23].

Considering the findings presented above, it’s crucial to adopt precise strategies/methods capable to specifically quantify α-syn neurotoxic species in biological samples. In this context, we demonstrated the development of specific, sensitive and reliable ELISA assays using our antibodies to assess different species of α-syn (t-, o- and p-S129-α-syn) in different biological samples. The provision of such reliable immunoassays is not only valuable for the discovery of ideal CSF biomarkers but also for a better understanding of the underlying pathophysiological process in PD and related disorders. Next, to explore the potential of our novel assays, we employed them to quantify different species of α-syn in CSF from a cohort of PD patients and age-matched healthy controls (HC). Finally, we investigated the usefulness of combining several biomarkers that reflect different pathogenic pathways, including AD classical biomarkers (total-tau (t-tau), phosphorylated-tau (p-tau) and amyloid-beta-42 (Aβ-42)), as candidate CSF biomarkers for PD and related disorders.

## Results

### Development and characterization of our anti-α-syn antibodies

**Fig. 1:**
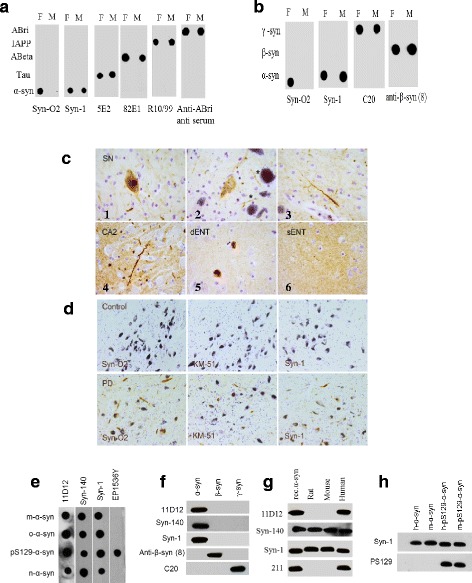
Characterization of anti-α-syn antibodies. 50 ng of monomers (M) and fibrils (F) of human α-syn, ABri, Islet amyloid polypeptide (IAPP), Abeta or tau (**a**), and β- or γ-syn (**b**) were spotted onto nitrocellulose membranes, and then probed with the indicated antibodies. **c** Representative photomicrographs illustrating histopathological features detected with Syn-O2 in substantia nigra pars compacta (SNpc), CA2 and deep layers of entorhinal cortex (dENT) or superficial layers of entorhinal cortex (sENT) of a PD patient (male, 84 years; Braak PD stage 6). 1) Lewy body and small bulgy neurites in SNpc; 2) neuron with punctated Syn-O2 immunoreactivity in SNpc. *Note the Syn-O2 immunoreactive beaded strings. 3) long thin threads and many small extracellular or synaptic Syn-O2 immunoreactivity; 4) a long thread in CA2 and many small neurites; 5) Lewy body pathology in deep layers of enthorinal cortex; 6) many small neurites and synaptic staining (Bar = 50 μm). **d** Photomicrographs showing immunoreactivity of Syn-O2, KM-51 and Syn-1 monoclonal antibodies in 10 μm thick paraffin-embedded sections of a control and PD patient. No staining was observed in the control with any of these mAbs. In PD patient, small and large LBs and LNs were observed with all three mAbs. With Syn-O2, many other long neurites and small extracellular or synaptic-like aggregates were stained. Bar = 50 μm. **e** Monomeric (m-α-syn), oligomeric (o-α-syn), phosphorylated α-syn (p-Ser129-α-syn) and nitrated (n-α-syn) forms were used to test the cross-reactivity of our antibodies 11D12 and Syn-140 · The generic commercial mAbs Syn-1 (BD Biosciences, 50 ng/ml) for α-syn, and EP1536Y (Abcam) for p-Ser129-α-syn, were also included as controls. **f** 50 ng of recombinant α-, β- and γ-syn were loaded on SDS gels and transferred to nitrocellulose membranes for western blotting, and then probed with our antibodies or control antibodies as appropriate. Syn-1 (BD Biosciences, 50 ng/ml) for α-syn, anti-β-synuclein (8) (Santa Cruz Biotechnology, 1:2 K) for β-syn and C-20 (Santa Cruz Biotechnology, 1:3 K) for γ-syn. **g** 15 μg of human, mouse and rat brain lysates were used in western blotting to test the specificity of 11D12 and Syn-140 · 50 ng of recombinant human α-syn (rec.α-syn) was included as positive control. **h** 50 ng of recombinant human α-syn (H-α-syn) or mouse α-syn (M-α-syn), human phosphorylated α-syn (H-p-Ser129-α-syn) and mouse phosphorylated α-syn (M-p-S129-α-syn) were loaded on SDS gels and transferred to nitrocellulose membranes for western blotting, and the membranes were then probed with our mouse mAb (PS129) recognizes both human and mouse p-Ser129-α-syn or the generic commercial mAb Syn-1 as indicated

Our mouse monoclonal antibody, Syn-O2, specifically recognizes early “soluble oligomers” and late aggregates “amyloid fibrils” of α-syn (Fig. 1a, b), and has a high binding affinity for o-α-syn (IC_50_ 120 pM and K_D_ 9.6 × 10^−11^ M) [24]. It exclusively bound to α-syn aggregates (amyloid fibrils and soluble oligomers) without cross-reactivity with monomers or fibrils generated from other amyloid proteins including β-syn, γ-syn, β-amyloid, tau, islet amyloid polypeptide (IAPP) or ABri (Fig. 1a, b). In immunohistochemical studies, Syn-O2 stained underappreciated small intra- and extracellular micro-aggregates and very thin neurites in PD and DLB cases that were not observed with generic pan antibodies (Syn-1 or KM51) that recognize linear epitope. Both classical Lewy neurites defined as elongated structures within neuronal processes, and Lewy neurites in the form of beaded strings; swollen/bulging neurites and long, thin threads were also stained by Syn-O2 (Fig. 1c, d) (for details see Additional file 1). The mAb,11D12, and a sheep polyclonal-antibody, Syn-140, both recognized different forms/species of α-syn including monomeric-, oligomeric-, protofibrillar-, nitrated- and phosphorylated-S129-α-syn, as well as full-length and N- or C-terminal truncated forms of α-syn (Fig. 1e-g). Finally, PS129, a mouse mAb was specific for p-S129-α-syn (Fig. 1h).

### Development of new ELISA assays for measuring different species of α-syn

**Fig. 2:**
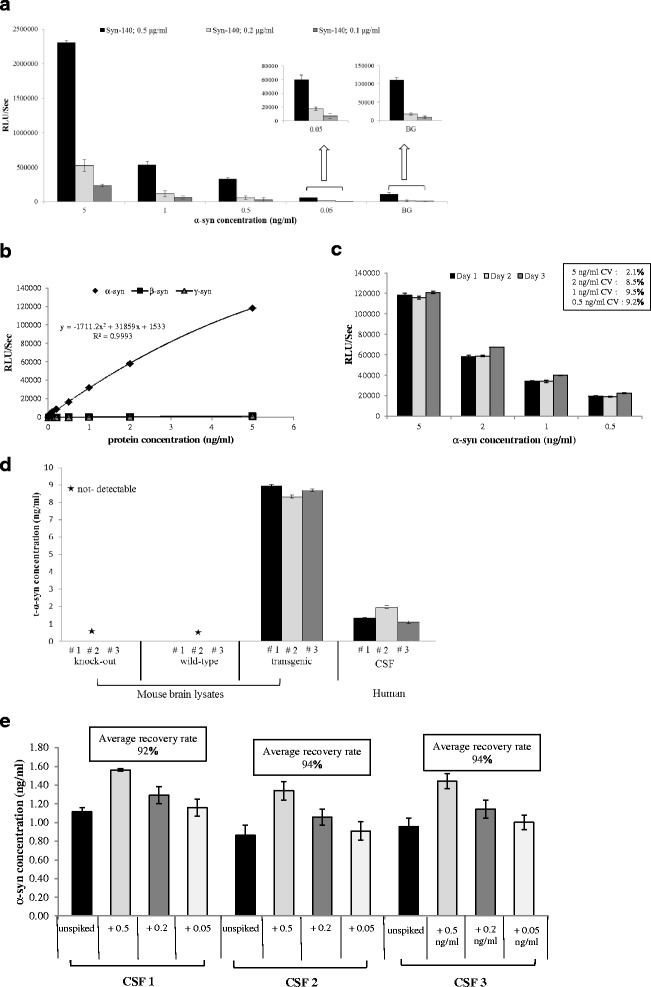
Validation of ELISA specific for total α-syn. **a** Histograms representing the optimization of the capture antibody (Syn-140) concentration. Based on the sensitivity and background signal (BG), 0.1 μg/ml of the capture antibody was selected for subsequent assays. **b** Standard curves displaying the specificities and sensitivities for α- (♦), β- (■) and γ-syn (▲). **c** Data shown are representative of 3 independent experiments using serial dilutions of recombinant α-syn freshly prepared over 3 non-consecutive days. Measurements were taken in duplicate, and the results show the mean ± standard deviation for each point, and the inter-assay coefficient of variation (CV) was < 10 %. **d** Antibody specificity determination using murine brain lysates and human CSF; Tg, WT and KO brain lysates were diluted to 5 μg/ml. Our assay permitted the direct quantification of native α-syn in biological samples such as human CSF and Tg mouse-brain lysates, whereas no signal was observed in WT or KO lysates. **e** Assessment of α-syn recovery rate in human CSF. The recovery rates were calculated from measured α-syn concentrations relative to spiked amounts, and the assay consistently featured a mean recovery rate > 90 %

We developed, validated and optimized a new sandwich-type ELISA for measuring t-α-syn levels in human CSF. Assay conditions were first optimized using different concentrations of the capture antibody Syn-140 (sheep anti-α-syn polyclonal antibody). Based on its sensitivity and background signal, 0.1 μg/ml of the capture antibody was selected for subsequent assays. The detection limit of the assay was as low as 50 pg/ml, which is 20-fold less than the concentration detected in human CSF (Fig. 2a). The standard curve for the ELISA assay was constructed using different concentrations of recombinant human α-syn in solution. Serial dilutions of recombinant human β-syn and γ-syn were also included as negative controls (Fig. 2b). The inter-assay coefficient of variation (CV) examined over >10 plates was < 10 % (Fig. 2c). As illustrated in (Fig. 2d), our assay permitted the direct quantification of native α-syn in biological samples such as human CSF and Tg mouse-brain lysates (for details see Additional file 1). α-Syn was detected in Tg mice and human CSF, whereas no signal was observed in WT or KO lysates (Fig. 2d). Moreover, recovery rates were determined by spiking recombinant α-syn into human CSF. Our assay consistently featured a mean recovery rate > 90 % (Fig. 2e).

**Fig. 3:**
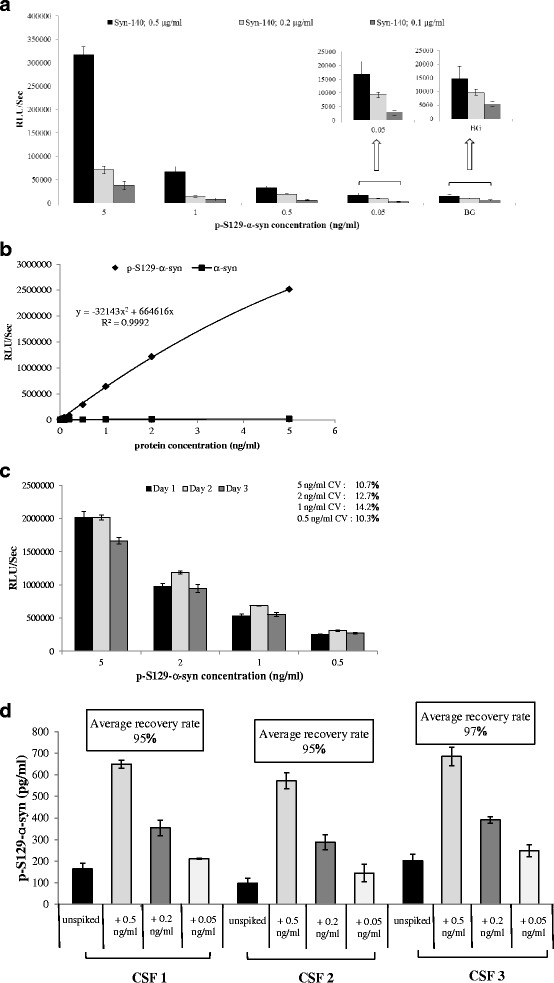
Validation of ELISA specific for p-S129-α-syn. **a** Standard curves displaying the specificities and sensitivities for p-S129-α-syn (♦) and non-phosphorylated α-syn (■). **b** Histograms representing the optimization of the capture antibody (Syn-140) concentration and the background signal (BG). **c** Examination of inter-assay variability. Measurements were taken in duplicate, and the results show the mean ± standard deviation for each point, and the inter-assay variability was found < 15 %. **d** Assessment of α-syn recovery rate in human CSF. The recovery rates were calculated between 95 and 97 % from measured α-syn concentrations relative to spiked amounts

We followed a similar protocol for p-S129-α-syn-specific ELISA, where Syn-140 was used as the capture antibody and PS129 was used as the reporter. To address the issue of specificity, serial dilutions of recombinant human p-S129-α-syn and non-phosphorylated α-syn were tested, and signal was detected only with the p-S129-α-syn (Fig. 3a). Using similar criteria (sensitivity, reproducibility and background signal), optimal conditions were adopted for the assay (Fig. 3b). The limit of detection was as low as 20 pg/ml, and a standard curve with R^2^ of 0.999 was generated (Fig. 3b), whereas, the inter-assay CV was < 15 % (Fig. 3c). To explore the assay suitability for quantifying p-S129-α-syn in biological fluids, multiple concentrations of recombinant human p-S129-α-syn were spiked into human CSF. The mean recovery rates were between 95 and 97 % (Fig. 3d).

**Fig. 4:**
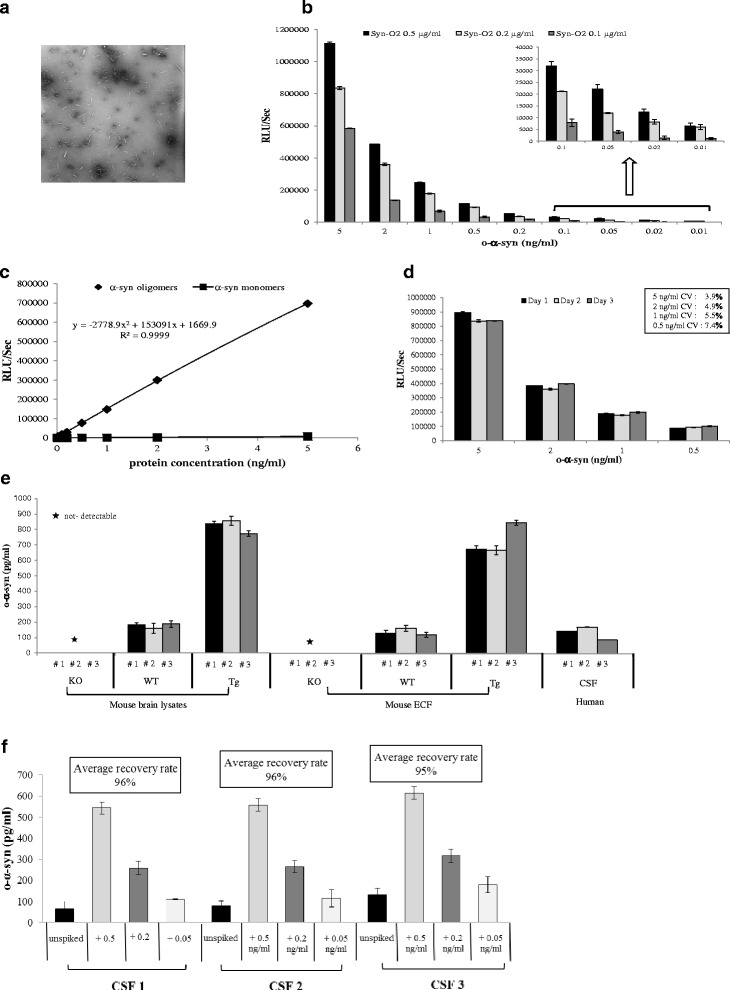
Validation of oligomeric-ELISA specific for α-syn oligomers. **a** Electron microscopy image of negatively stained sample of α-syn solution containing soluble oligomers and protofibrils which was used as the ELISA standard. **b** Standard curves displaying the specificities and sensitivities for α-syn oligomers (♦), and α-syn monomers (■). **c** Histograms representing the optimization of the capture antibody (Syn-O2) concentration. **d** Assessment of inter-assay variability. Measurements were taken in duplicate, and the results show the mean ± standard deviation for each point, and the intra-assay and inter-assay analysis revealed a CV of < 10 %. **e** Detection of o-α-syn in human CSF, Tg, WT and KO brain lysates. O-α-syn levels were significantly greater in Tg mice compared with WT, whereas no signal was detected in KO mice. The assay also quantified successfully the levels of o-α-syn in human CSF. **f** Assessment of o-α-syn recovery rate in human CSF. Recovery rates were calculated from measured o-α-syn concentrations relative to spiked amounts and the average recovery rates were > 95 %

Following the complete characterization of our conformation-specific monoclonal antibody Syn-O2 as described above, we developed and optimized novel specific ELISA for measuring o-α-syn in human CSF. Similarly, our oligomeric ELISA is based on a conventional sandwich ELISA system, where o-α-syn is captured using our highly specific anti-o-α-syn mAb; Syn-O2. Multiple conditions were tested to assess assay performance. Serial dilutions of recombinant human o-α-syn were run in duplicates. Based on optimal sensitivity, specificity, reproducibility and background signal results, we selected a concentration of 0.2 μg/ml for coating (Fig. 4b). Our assay specificity towards o-α-syn was further validated as no signal was detected when serial dilutions of α-syn monomers were included (Fig. 4c). The intra-assay and inter-assay precision was < 10 % (Fig. 4d). To assess the specificity and sensitivity of our oligomeric-ELISA for o-α-syn in biological samples, brain lysates and microdialysis fluid from young Tg mice that over-expressed human α-syn were used, and WT and KO mice were also included. As anticipated, o-α-syn levels were significantly greater in Tg mice compared with WT, whereas no signal was detected in KO mice (Fig. 4e). In parallel, our assay quantified the levels of o-α-syn in human CSF successfully (Fig. 4e). Next, we spiked increasing amounts of recombinant o-α-syn into human CSF samples. Interestingly, the average recovery rates were > 95 % (Fig. 4f). We estimated that the lower limit of detection of recombinant o-α-syn using this ELISA was as low as 10 pg/ml based on the initial concentration of the protein (5 ng/ml). Taken together, the results show that our oligomeric-ELISA is specific, sensitive and reproducible, and it is thus suitable for the quantification of o-α-syn in biological samples.

### CSF biomarkers in diagnostic groups

We then measured the levels of different α-syn species in human CSF of an independent cross-sectional cohort consisting of individuals diagnosed with PD (Table 1) and age-matched HC. Consistent with previous reports [11, 14, 16, 22], CSF t-α-syn levels were significantly decreased in PD compared to HC (*p* < 0.0001), whereas, o-α-syn levels were significantly higher in the PD group (*p* < 0.0001). CSF p-S129-α-syn levels were elevated in PD compared to HC, with considerable overlap between the groups (*p* <0.001). However, both p-S129-/t-α-syn and o-/t-α-syn ratios improved the discrimination of PD from HC (*p* < 0.0001). CSF t-tau, p-tau and Aβ42 levels did not differ significantly between PD and HC groups (Table 1).

**Table 1:** Demographics, clinical features and biomarkers

	HC (*n* = 48)	PD (*n* = 46)	*p*-values
Female	32 (66.6 %)	18 (39 %)	0.85
Male	16 (33.3 %)	28 (61 %)	
Age	63 (57–67)	64 (57–71)	0.7969
Disease years	-	4 (2–8.3)	-
H&Y Stage	-	2 (2–2.5)	N.A
UPDRS- III	-	23 (15.3–27)	N.A
MMSE	29 (29–30)	29 (28–30)	0.0479
t-α-syn (ng/ml)	1.6 (1.3–2.2)	1.3 (1.2–1.6)	<0.0001
p-S129-α-syn (pg/ml)	222 (180.5–275)	261 (206.8–296.3)	<0.001
o-α-syn (pg/ml)	57 (36–106.5)	116 (76–170)	<0.0001
p-S129/t-α-syn %	13.7 (9.2–18.5)	18.6 (15.7–23.3)	<0.0001
o-/t-α-syn %	3.5 (2.3–6.2)	8.9 (5.2–12.2)	<0.0001
t-tau (pg/ml)	229 (162–271.5)	190 (157.8–274.3)	0.1710
p-tau (pg/ml)	41.5 (29.3–50.3)	41.1 ± 16	0.5965
Aβ42 (pg/ml)	995.5 (877.5–1153)	966.5 (794–1077)	0.6745

Median, interquartile ranges and p-values of Mann-Whitney *U* test continuous variables, Count (percentage) for categorical ones

Abbreviations: *HC* healthy controls, *PD* Parkinson’s disease, *H&Y* Hoehn and Yahr, *UPDRS- III* Unified Parkinson’s Disease Rating Scale-Part-III, *MMSE* mini-mental state examination, *t-α-syn* total α-synuclein, *p-S129-α-syn* phosphorylated Ser 129 α-synuclein, *o-α-syn* oligomeric α-synuclein, *t-tau* total-tau, *p-tau* phosphyorylated-tau, *Aβ42* amyloid beta-42

### Cross sectional correlation analysis of α-syn species in CSF

**Fig. 5:**
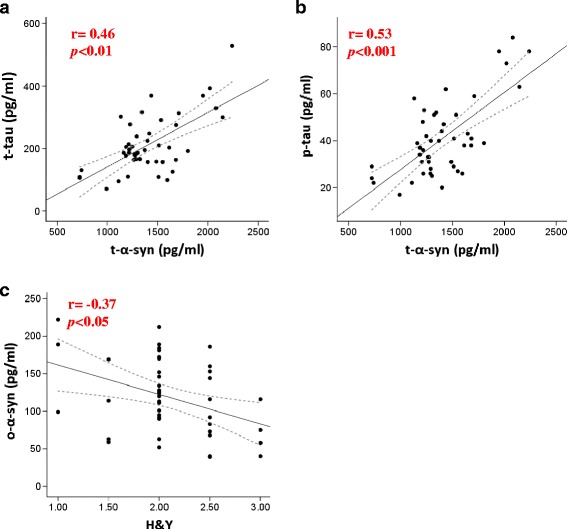
Scatter plots showing the correlation between t-α-syn and t-tau, p-tau and between o-α-syn and Hoehn and Yahr score (H&Y) scores. The correlation between CSF t-α-syn with t-tau (*r* =0 · 46, *p* < 0 · 01) and p-tau (*r* = 0 · 53, *p* < 0 · 001) in the PD group (**a-b**). The correlation between CSF o-α-syn with H&Y scores (*r* = −0 · 37, *p* < 0 · 05) in the PD group (**c**). The dotted line is the 95 % prediction interval for the calculated regression line (solid line)

A positive correlation with t-α-syn was found for both t-tau and p-tau (r_s_ = 0.47, r_s_ = 0.53 respectively, *p <* 0.00, Fig. 5a, b) in the PD group. In contrast, an inverse significant correlation was observed between p-S129-syn levels and p-tau (r_s_ = −0.30, *p* =0.043) within the PD group. The levels of CSF o-α-syn did not correlate with any of AD biomarkers (Table 2). While CSF t-α-syn did not correlate with H&Y, UPDRS-III or disease duration, an inverse correlation with MMSE scores was observed (r_s_ = −0.46, *p* < 0.001) within the PD group (Table 3). Similarly, CSF p-S129-α-syn did not correlate with disease severity. On the other hand, a significant inverse correlation was noted between CSF o-α-syn and H&Y scores (r_s_ = −0.37, *p* = 0.015, Fig. 5c) within PD group, but not with UPDRS, disease duration or MMSE scores. As anticipated, t-tau and p-tau positively correlated with age (*p* = 0.003, *p* < 0.0001, respectively) and negatively correlated with MMSE scores in the PD group (r_s_ = −0.35, p = 0.01, r_s_ = −0.38, p = 0.009 respectively), whereas, Aβ42 didn’t correlate significantly with any of the clinical parameters.

**Table 2:** Spearman correlations between CSF biomarkers in PD

	t-α-syn	p-α-syn	o-α-syn	t-Tau	p-Tau	Aβ42
t-α-syn	1 · 00					
p-S129-α-syn	0 · 002	1 · 00				
o-α-syn	0 · 21	−0 · 03	1 · 00			
t-Tau	0 · 46**	−0 · 19	0 · 06	1 · 00		
p-Tau	0 · 53***	−0 · 30*	0 · 14	0 · 86**	1 · 00	
Aβ42	0 · 09	−0 · 14	0 · 5284	0 · 31*	-	1 · 00

Abbreviations: *t-α-syn* total α-synuclein, *p-S129-α-syn* phosphorylated Ser 129 α-synuclein, *o-α-syn* oligomeric α-synuclein, *t-tau* total-tau, *p-tau* phosphyorylated-tau, *Aβ42* Amyloid Beta-42. (*p <0.05, **p <0.01, ***p <0.001)

**Table 3:** Spearman correlations between CSF biomarkers, disease duration (years), UPDRS-III, Hoehn and Yahr stage and MMSE in PD

	Disease years	UPDRS-III	Hoehn and Yahr stage	MMSE
t-α-syn	0 · 06	0 · 12	0 · 18	−0 · 46**
p-S129-α-syn	0 · 04	−0 · 16	−0 · 18	−0 · 05
o-α-syn	−0 · 25	−0 · 19	−0 · 37*	−0 · 01
p-S129/t-α-syn %	−0 · 16	−0 · 22	0 · 002	0 · 39**
o/t-α-syn %	−0 · 25	−0 · 26	−0 · 31*	0 · 24

Abbreviations: *t-α-syn* total α-synuclein, *p-S129-α-syn* phosphorylated Ser 129 α-synuclein, *o-α-syn* oligomeric α-synuclein, *UPDRS- III* Unified Parkinson’s Disease Rating Scale-Part-III, *H&Y* Hoehn and Yahr, *MMSE* mini-mental state examination. (*p <0.05, **p <0.01, ***p <0.001)

### Logistic regression analysis

**Fig. 6:**
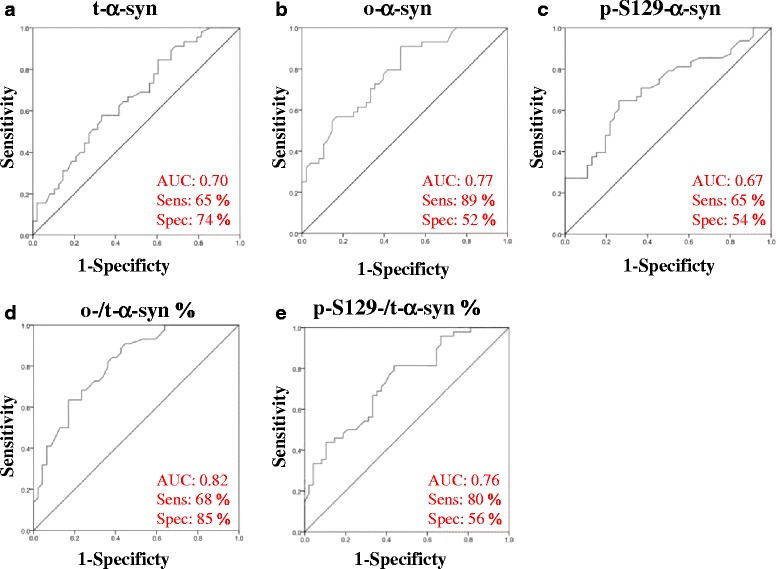
Use of receiver operating curves (ROC) for the levels of α-syn species in CSF. ROC curves based on logistic regression analyses for the classification of PD patients versus HC based on various predictors and combination of predictors. (**a**) t-α-syn, (**b**) o-α-syn, (**c**) p-S129-α-syn, (**d**) o-/t-α-syn% and (**e**) p-S129-/t-α-syn%

Binary logistic regression analysis revealed five CSF biomarkers as individual PD predictors: t-α-syn (*p* = 0.003), o-α-syn (*p* <0.0001), p-S129-α-syn (*p* < 0.0001), o-/t-α-syn ratio (*p* <0.0001) and p-S129/t-α-syn ratio (*p* < 0.0001; Fig. 6a-e). The combination of o-/t-α-syn, p-S129-α-syn and p-tau formed the best fitting predictive model for discriminating PD patients from controls (Table 4). The sensitivity and specificity of the combination of o-/t-α-syn ratio, p-S129-α-syn and p-tau was 79 % and 67 % (AUC = 0.86). The performance of this predictive model was significantly better compared to o-/t-syn ratio, p-S129-syn alone (See Table 4 for further details).

**Table 4:** Logistic regression analysis of CSF biomarkers between PD and HC

Predictor	AUC	Sensitivity	Specificty	*p* value
t-α-syn	0 · 70	65 %	74 %	0 · 001
p-S129-α-syn	0 · 67	65 %	54 %	<0 · 0001
o-α-syn	0 · 77	89 %	52 %	< 0 · 0001
p-S129/t-α-syn %	0 · 76	80 %	56 %	< 0 · 0001
o-/t-α-syn %	0 · 82	68 %	85 %	< 0 · 0001
o-/t-α-syn %, p-S129-α-syn & p-tau	0 · 86	79 %	67 %	< 0 · 0001

Abbreviations: *t-α-syn* total α-synuclein, *p-S129-α-syn* phosphorylated Ser 129 α-synuclein, *o-α-syn* oligomeric α-synuclein, *p-tau* phosphorylated-tau, *AUC* area under the curve

## Discussion

The lack of reliable biomarkers is a major obstacle holding us back from accurately diagnosing PD or monitoring its progression. Since the precise identification of toxic α-syn species is still unclear, we aimed to generate antibodies capable to detect wide range of the different α-syn species including the pathogenic species o-α-syn and p-S129-α-syn and then utilized these antibodies to develop total-, oligomeric- and p-S129-ELISA systems capable to quantify specifically these species in different biological samples. Our novel antibody, Syn-O2, can facilitate our ability to study o-α-syn and explore its toxic characteristics. Moreover, Syn-O2 could provide insights into the possible strategies through which we can halt PD progression or reverse o-α-syn toxicity. Applying different immunoassays, Syn-O2 was shown to be highly selective for o-α-syn since it did not cross-react with aggregated forms of other amyloidogenic proteins (tau, Abeta, IAPP and ABri) and it did not recognize the other synuclein proteins (β- or γ-syn). Moreover, Syn-O2 clearly stained LBs and LNs only. Most interestingly, comparing Syn-O2 staining pattern with the other commercial antibodies that are widely used in IHC (Syn-1, KM-51), Syn-O2 not only showed a lack of cross-reactivity with synaptic α-syn, but also detected pathology not detectable by other pan antibodies confirming its specificity toward α-syn pathology.

Both Syn-140 and 11D12 are specific for α-syn, and while Syn-140 recognizes α-syn from different species (human, mouse and rat), 11D12 is specific only for human α-syn. Moreover, PS129 specifically recognizes p-S129-α-syn and does not cross-react with non-phosphorylated α-syn. This diversity in the specifications/characteristics of our antibodies imparts distinctive virtues to our ELISA assays. Therefore, our assays can serve as powerful research tools to investigate the potential of α-syn species in different biological samples. Furthermore, our new ELISA assays provide multiple improvements over other reported immunoassays. First, our ELISA design using 384-well plates is perfectly compatible for accommodating multiple replicates even with limited volume of the sample (50 μl/well). Second, the enhanced sensitivity shown by our assays validates their suitability for the analysis of human CSF specimens. Our assays were shown to be highly target specific based on several methods, including specificity validation of the employed antibodies to their respective antigens, using brain and blood products from genetically modified mice (Tg, WT, KO) and monitoring assay precisions. Our ELISAs are also robust since recovery rates from spike experiments were > 90 %.

Our group has previously described the development of an ELISA for quantifying o-α-syn in human CSF using the same mouse mAb (211, mouse anti-α-syn antibody, Santa Cruz) for capture and the biotinylated form of 211 for detection [14, 25]. However, heterophilic antibodies may interfere in this assay format when using the same antibody for capturing and detection, leading to false-positive signals [26]. Our new oligomeric-ELISA has addressed this shortcoming by using our novel mouse conformation-specific mAb (Syn-O2), which selectively captures o-α-syn, and a rabbit polyclonal antibody (FL-140) for detection, thus permitting the precise quantification of o-α-syn in biological samples. Furthermore, our new oligomeric-ELISA system showed great sensitivity and specificity for measuring α-syn oligomers in biological samples in comparison to our previous oligomeric-ELISA system.

Analyses of the cross sectional cohort revealed that while concentrations of t-α-syn decreased, o-α-syn concentrations significantly increased in PD compared to HC, consistent with other studies [11, 12, 16, 27–29]. The reduction in CSF t-α-syn is likely due to α-syn oligomerisation and sequestration in LBs [11, 30], and we speculate that the elevated levels of o-α-syn results from a clearance failure of the aggregated forms of α-syn. It’s worth mentioning, that in a recent study by Compta et al. [29], the levels of o-α-syn were determined using our previously published oligomeric-ELISA [14], where o-α-syn levels were found to be elevated in PD patients consistent with our findings in this study. However, we attribute the differences in the levels between both studies to the use of different quantification methods, pre-analytical confounding factors (types of tubes, spinning conditions, storage conditions, etc.) and differences in disease severity between the patients included in the studies. Furthermore, as emphasized above, we found our new oligomeric-ELISA to be more reliable compared to the old generation oligomeric-ELISA in terms of sensitivity, specificity and robustness.

PD patients with dementia were not enrolled in our cohort, as reflected in the overall high MMSE scores registered. These high MMSE scores could explain the weakness of the correlation between MMSE scores and t-α-syn levels and the lack of a correlation with Aβ42 levels. Interestingly, CSF o-α-syn levels showed modest negative correlation with disease motor severity (H&Y grade). This inverse correlation, although weak, suggests that o-α-syn increases at the early stage of the disease prior to the manifestation of the motor symptoms. Interestingly, similar finding has been recently reported in sporadic PD patients and PD cases with leucine-rich repeat kinase 2 mutations [23].

An increase in p-S129-α-syn levels was also observed in the PD group compared to HC. Although, approximately 90 % of accumulated α-syn in LBs consists of p-S129-α-syn [31], it is not known whether phosphorylation of α-syn promotes or prevents the formation of toxic α-syn aggregates. To date there is only one reported study, in which there was a weak positive correlation between CSF p-S129-α-syn/t-α-syn ratio and disease severity [15], however we did not observe such relationship in our current study. These discrepancies are likely due to several factors, including differences in the assay platform (Luminex versus sandwich-ELISA), collection and handling of the CSF samples and/or heterogeneity of the patients included in the studies.

Nevertheless, including both o-/t-α-syn and p-S129-/t-α-syn ratios improved the ability to discriminate between PD and HC, this was further enhanced by including p-tau, emphasizing the usefulness of combining several CSF biomarkers for diagnostic purposes.

Synucleinopathies and tauopathies show significant clinical overlap, making the early diagnosis of PD more challenging. This overlap necessitates a combination of measurements of CSF α-syn species with key AD biomarkers to improve diagnostic accuracy. Moreover, several in vitro and in vivo studies investigated interactions among α-syn and tau proteins, showing that these proteins promote each other’s aggregation, leading to neuronal degeneration and worsening cognitive impairment [32]. The positive correlation between CSF t-α-syn and t-tau and p-tau in the PD group we observed, confirms and extends reports by others showing a positive correlation between CSF t-α-syn and tau levels in PD, AD and controls [33].

## Conclusions

In summary, using our novel mAbs, we have developed sensitive and specific ELISAs to measure t-, o- and p-S129-α-syn species in human CSF. Combining measurements of different α-syn species in CSF, we observed marked differential CSF patterns between PD and controls. Our results validate the usefulness of combining multiple CSF biomarkers in improving PD diagnostic accuracy and prognosis. Although the potential use of our assays was confirmed in an independent cohort, we still need to validate the utilization of our assays in large-scale, prospective, longitudinal and well-controlled studies such as the ongoing Parkinson’s Progression Markers Initiative, and by inclusion of patients with other neurological illnesses.

## Methods

### Generation of anti-α-syn antibodies

Generation and purification of our mouse monoclonal antibodies were performed as previously described [24]. Briefly, the antigens, recombinant human α-syn protein, α-syn fibrils [34] or synthetic phosphopeptide-S129 (CYEMPSEEGY (α-syn aa 125 to 133; [35, 36]) were used to generate 11D12, Syn-O2 and PS129 mouse monoclonal antibodies respectively. The antigens were repeatedly used to immunize female BALB/C mice subcutaneously. Mice with appropriate plasma titers were euthanized and splenocytes were extracted and fused with Sp2/0 myeloma cells and then seeded in HAT (hypoxantin, aminopterine, and thymidine) selective medium to generate antibody-producing hybridomas according to standard techniques. After 10 days, the medium was replaced with hypoxantin, thymidine medium and 5 days later the culture supernatants were tested for secreted anti-α-syn antibodies using indirect ELISA. Several monoclonal antibodies were produced, purified and thoroughly characterized, among which Syn-O2 (IgG1), 11D12 (IgG2a) and PS129 (IgG2a) were selected for this study. The isotypes were determined using an isotyping kit (Sigma-Aldrich, USA) and the antibody purification was done using Protein G-agarose (Sigma-Aldrich, USA) affinity chromatography.

Syn-140 is a sheep polyclonal antibody raised against full-length human α-syn as previously described [37, 38]. In brief, a polyclonal antiserum was raised against α-syn in sheep by intramuscular injection into four sites of 500 μg recombinant α-syn protein emulsified in Freund’s complete adjuvant. Boosts of 200 μg in Freund’s incomplete adjuvant were given intramuscularly into four sites at intervals after the primary injection of 1 month, 2 months and 6 months. The sheep were bled 8 days after the final boost.

### Dot blot

Monomeric and aggregated forms of recombinant α-, β- and γ-syn or tau proteins and synthetic peptides Aβ-42 [9, 39]; Islet Amyloid Polypeptide (IAPP) [40]; or ABri [35] were used for testing the specificity of Syn-O2. Each protein/peptide was spotted (50 ng) onto a nitrocellulose membrane and allowed to dry at RT for 1 h. The membranes were then incubated for 1 h at RT with 5 % skimmed milk in PBST (PBS containing 0.05 % Tween-20) with gentle agitation. The blocking solution was then removed, and the membranes were incubated for 2 h at RT with the primary antibodies: Syn-O2; mouse anti-α-syn (Syn-1, BD Bioscience); mouse anti-β-syn (8; Santa Cruz Biotechnology); goat anti-γ-syn (C-20, Santa Cruz Biotechnology); mouse anti-tau (5E2, Millipore); mouse anti-amyloid beta (82E1, IBL) or mouse anti-amylin (R10/99, Santa Cruz Biotechnology). The membranes were then washed three times with PBST and incubated with secondary antibodies, goat anti-mouse HRP (1:20 K, Jackson Immunoresearch, PA, USA); goat anti-rabbit (1:20 K, Jackson Immunoresearch, PA, USA) or chicken anti-goat HRP (1:30 K, Santa Cruz Biotechnology) as appropriate for 1 h at RT. The membranes were then washed three times with PBST and developed using SuperSignal West Pico Chemiluminescent Substrate (Pierce).

### Western blotting

Samples of human-, mouse- and rat-brain lysates (15 μg), recombinant α-, β- and γ-syn or human and mouse p-S129-α-syn (50 ng) were mixed with loading buffer (250 mM Tris-HCl, pH 6.8, 30 % glycerol, 0.02 % bromophenol blue) and then separated on 15 % SDS-PAGE gels. The separated proteins were transferred to 0.45 μm nitrocellulose membranes (WhatmanGmbh-Germany) at 100 V for 45 min. The membranes were boiled for 5 min in PBS and then blocked for 1 h with 5 % non-fat milk prepared in PBST prior to incubation with 10 ml of primary antibody (50 ng/ml) for 2 h at RT. The membranes were then washed 4 times with PBST followed by incubation with the secondary antibody. After one hour, the membranes were washed 4 times with PBST, and immunoreactive bands were visualized using the SuperSignal West Pico Chemiluminescent Substrate Kit (Pierce) according to the manufacturer’s instructions.

### Preparation of the standard for the oligomeric ELISA

Expression and purification of recombinant human α-syn was performed as previously described [34]. α-Syn oligomers were prepared by incubating fresh α-syn (25 μM) with Ginsenoside Rb1 (100 μM) in PBS at 37 °C for 5 days with continuous shaking at 800 rpm in a Thermomixer (Eppendorf), then α-syn oligomers were characterized using electron microscopy as previously described [41]. Briefly, size exclusion chromatography was carried out using an AKTA FPLC system (GE Healthcare, Sweden) and a Superdex 200 column at 4 °C, in order to separate soluble α-syn oligomers from monomers. This way, we guaranteed that only pure soluble α-syn oligomers were separated and then confirmed by electron microscopy (Fig. 4a) [41].

### Development of ELISA for measuring t-α-syn in human CSF

A 384-well ELISA microplate (Nunc MaxiSorb, NUNC) was coated by overnight incubation at 4 °C with 0.1 μg/ml Syn-140 (sheep anti-α-syn polyclonal antibody) in 200 mM NaHCO_3_, pH 9.6 (50 μl/well). The plate was then washed with PBST and incubated with 100 μl/well of blocking buffer (PBST containing 2.5 % gelatin) for 2 h at 37 °C. After washing, 50 μl of the CSF samples (thawed on ice and Tween-20 was added to a final concentration of 0.05 %) was added to each well, and plates were incubated at 37 °C for 2.5 h. 11D12 (mouse anti-α-syn monoclonal antibody), diluted in blocking buffer at 1:5 K was added to the appropriate wells, and incubated at 37 °C for 2 h. Next, the plate was washed and incubated for 2 h at 37 °C with 50 μl/well of species-appropriate secondary antibody (donkey anti-mouse IgG HRP, Jackson ImmunoResearch) diluted in blocking buffer (1:20 K). After washing, the plate was incubated with 50 μl/well of an enhanced chemiluminescent substrate (SuperSignal ELISA Femto, Pierce Biotechnology, Rockford, IL). The chemiluminescence, expressed in relative light units, was immediately measured using VICTOR™ X3 multilabel plate reader (PerkinElmer). The standard curve for the ELISA assay was carried out using serial dilutions of recombinant human α-syn in artificial CSF.

### Development of ELISA for measuring p-S129-α-syn in human CSF

A 384-well ELISA microplate (Nunc MaxiSorb, Nunc) was coated by overnight incubation at 4 °C with 0.5 μg/ml Syn-140 (sheep anti-α-syn polyclonal antibody) in 200 mM NaHCO_3_, pH 9.6 (50 μl/well). The plate was then washed with PBST and incubated with 100 μl/well of blocking buffer for 2 h at 37 °C. After washing, 50 μl of the CSF samples (thawed on ice and Tween-20 was added to a final concentration of 0.05 %) was added to each well, and plates were incubated at 37 °C for 2.5 h. PS129 (mouse anti-pS129-α-syn monoclonal antibody) diluted in blocking buffer (1:1 K) were added to the appropriate wells, and incubated at 37 °C for 2 h. Next, the plate was washed and incubated for 2 h at 37 °C with 50 μl/well of species-appropriate secondary antibody (donkey anti-mouse IgG HRP (Jackson ImmunoResearch)) diluted in blocking buffer (1:20 K). After washing, the plate was incubated with 50 μl/well of an enhanced chemiluminescent substrate (SuperSignal ELISA Femto, Pierce Biotechnology, Rockford, IL). The chemiluminescence, expressed in relative light units, was immediately measured using VICTOR™ X3 multilabel plate reader (PerkinElmer). The standard curve for the ELISA assay was obtained using serial dilutions of recombinant human p-S129-α-syn in artificial CSF (50 μl/well).

### Development of ELISA for measuring o-α-syn in human CSF

A 384-well ELISA microplate (Nunc MaxiSorb, Nunc) was coated by overnight incubation at 4 °C with Syn-O2 (0.2 μg/ml) in 200 mM NaHCO_3_, pH 9.6 (50 μl/well). The plate was then washed with PBST and incubated with 100 μl/well of blocking buffer for 2 h at 37 °C. After washing, 50 μl of the CSF samples (thawed on ice and Tween-20 was added to a final concentration of 0.05 %) was added to each well, and plates were incubated at 37 °C for 2.5 h. FL-140 (rabbit polyclonal antibody, Santa Cruz Biotechnology, Santa Cruz, CA, USA), diluted in blocking buffer at 1:1 K, was added to the appropriate wells, and incubated at 37 °C for 2 h. Next, the plate was washed and incubated for 2 h at 37 °C with 50 μl/well of goat anti-rabbit IgG HRP (Jackson ImmunoResearch) diluted in blocking buffer (1:15 K). After washing, the plate was incubated with 50 μl/well of an enhanced chemiluminescent substrate (SuperSignal ELISA Femto, Pierce Biotechnology, Rockford, IL). The chemiluminescence, expressed in relative light units, was immediately measured using VICTOR™ X3 multilabel plate reader (PerkinElmer). The standard curve for the ELISA assay was obtained using serial dilutions of recombinant human o-α-syn in artificial CSF (50 μl/well).

### AD CSF biomarkers

Concentrations of t-tau, p-tau and Aβ42 in CSF were determined using the sandwich ELISAs Innotest β-Amyloid (1–42), Innotest h TAU-Ag™ and Innotest Phosphotau (181P)™; (Fujirebio Diagnostics), respectively, as described previously [42].

### Study population

A description of this cross-sectional cohort has been previously published [27]. We included 46 patients with PD that attended the outpatient clinic for movement disorders at the VU University Medical Center (VUMC) between September 2008 and February 2011, and 48 self-declared age-matched HC who were recruited via an advertisement in the periodical of the Dutch Parkinson Foundation. All patients with PD fulfilled the United Kingdom Parkinson’s Disease Society Brain Bank clinical diagnostic criteria [43]. Patients were included only if they were able to understand the study aim and procedures. Mini-Mental State Examination and/ or neuropsychological assessment in the patients did not indicate dementia. In the controls, dementia was excluded using the Cambridge Cognitive Examination scale [44]. Patients and controls underwent a standardized clinical assessment that included their medical history and a neurological examination. Severity of parkinsonism and disease stage in the ‘on’ state were rated using the Unified Parkinson’s Disease Rating Scale-Part-III (UPDRS-III) [45] and the modified Hoehn and Yahr (H&Y) classification [46], respectively. All the samples were screened in a blinded fashion and were tested randomly. All the results were confirmed using at least two independent experiments. A series of internal controls were also run to check for run-to-run variations. All CSF samples with > 500 erythrocytes/μl were excluded from analysis as traces of blood may influence CSF α-syn levels [47].

### Data analysis

Statistical analyses were performed using using GraphPad Prism (version 5.0) software and IBM SPSS software 21. Continuous variables were described using medians and interquartile ranges. Categorical variables were presented as count or percentages. Mann-Whitney *U*-test was used for comparisons between PD and HC diagnostic groups. Correlations were calculated using Spearman’s Rho (r). Subjects with PD and HC were matched for age (*p* = 0 · 002), but not for gender since no significant differences between CSF biomarkers in males and females were found in PD or HC groups (*p* = 0 · 85). The accuracy of the diagnostic value of the biomarkers was assessed based on the area under the curve (AUC) of the receiver operating characteristic (ROC) curve [48]. Cut-off values were calculated using sensitivity and specificity values that maximized Youden’s index (sensitivity + specificity − 1). To determine the diagnostic value of a combination of multiple biomarkers, we performed a binary logistic regression analysis. Combinations of biomarkers were tested to find the best fitting model in a forward stepwise mode, which was based on the change in likelihood resulting from including the variable. Change in -2 log-likelihood statistics between the models was tested using Chi-square (*χ*2) tests. Sensitivity and specificity values of the combinations of biomarkers for PD diagnosis and progression values were also calculated.

## Additional file

Additional file 1: **Supplementary Materials and Methods.** (PDF 401 kb)
