# Supplementary material for: Oligomeric and phosphorylated alpha-synuclein as potential CSF biomarkers for Parkinson’s disease
Source: Mol Neurodegener. 2016 Jan 19;11:7. doi: 10.1186/s13024-016-0072-9 (PMC4717559; doi:10.1186/s13024-016-0072-9)
Supplement: Supplementary file 1 — Supplementary Materials and Methods. (PDF 401 kb) [file 13024_2016_72_MOESM1_ESM.pdf]

Materials and Methods

Post-mortem human brain tissue

Human post-mortem brain tissue was obtained from donors recruited by the Netherlands Brain Bank (NBB, Amsterdam, The Netherlands) and the pathology department of the VU University Medical Center (VUMC, Amsterdam, The Netherlands). In compliance with local ethical and legal guidelines, informed consent for their brain autopsy and use of brain tissue, neuropathological and clinical information for research purposes was given by either the donor or the next of kin. Extensive neuropathological assessment of disease-relevant brain regions was provided by established and qualified neuropathologists (JMR and WK). The density and distribution of LBs and LNs pathology was determined based on immunohistochemical analysis of α-syn (Clone KM51, Novastra, Bioconnect BV; dilution 1:500, pretreatment in citrate buffer pH 6.0 in the microwave and 80% formic acid) and Braak staging protocol [1]. Furthermore, the density of neurofibrillary tangles (NFT) and Aβ were determined based on Bodian Silver staining and immunohistochemical analysis of hyperphosphorylated tau (Clone AT8, Pierce, Rockford, IL) and Aβ (Clone 6F/3D, DAKO, DakoCytomation BV) throughout the brain using the criteria provided by Braak[2] and Brain Net Europe [3, 4].

Substantia nigra and hippocampus tissue of 12 clinically diagnosed and pathologically confirmed idiopathic PD or PD with dementia (PDD) patients (Braak PD stage 4-6) and 10 aged-matched controls were included in this study. Only idiopathic PD cases were included with an age of onset > 45 years and no history of other disorders of the central nervous system. The control group consisted of healthy subjects without neurological or psychiatric disease and without LB pathology (HC; Braak PD stage 0). All donors were carefully selected based on their

pathological assessment and clinical records. Furthermore, all subjects were controlled and matched for postmortem delay and cerebrospinal fluid pH value.

Tissue processing and immunohistochemistry

Brain tissue collected at autopsy was immersion-fixed in 4% formaldehyde for four weeks and subsequently disease-relevant areas were dissected and embedded in paraffin. Tissue samples were cut in 10 μm thick sections with a microtome, mounted on positively-charged glass slides (Menzel-Glaser SuperFrost Plus, Braunschweig, Germany) and dried in a stove overnight at 37°C. Antigen retrieval consisted of steaming in citrate buffer pH 6.0 and 5% Bovine albumin (BSA) in TBS -triton (TBS-Tx) served as blocking. Transverse sections of the mesencephalon at the level of the oculomotor nerve including the SN and temporal cortex including the hippocampus and parahippocampal gyrus sections were incubated with the primary antibody Syn-O2 using a dilution of 1:5K overnight at 4°C. EnVisiontm visualisation system (DAKO) was used to detect the primary antibody. EnVisiontm contains both the secondary antibodies for anti-mouse lgG specificity as well as a horseradish peroxidise (HRP)-conjugated polymer backbone. The dark brown chromogenic response was generated using 3, 3'-diaminobenzidine (DAB) peroxidise (Sigma-Aldrich) which reacts with the HRP enzyme label. Sections were counterstained using cresyl violet (Nissl) to visualize neurons and layers. A subset of sections was pretreated with proteinase K which is known to eliminate all non-accumulated, monomeric, soluble α-syn [5]. Adjacent sections were incubated with antibodies against α-syn (Clone KM-51, Novacastra, dilution 1:500; Syn-1, BD Biosciences, dilution 1:2K) for comparison of staining patterns.

Transgenic mice description

Approximately 7 kb of the mouse α-syn promoter was combined with human α-syn cDNA and the SV40 poly-adenylation sequence in a construct used to establish transgenic mouse lines by pronuclear microinjection into oocytes from C57Bl6xD2 hybrids. The subsequent transgenic lines were bred into C57Bl6 mice for at least nine generations. Non-transgenic mice from the same colony served as controls. Expression of the transgene was assessed by Northern blotting, in situ hybridization and Western blotting (Syn-1) using standard methods [6].

***SNCA*-null mice:**

KO mice were created by replacing a 400 bp genomic fragment containing the translocation start with a neocassette. WT mice in which the fragment was not knocked out were also used as controls [6].

Brain lysates preparation

Frozen brain samples (1 g) from transgenic (Tg), wild-type (WT) and knock-out (KO) mice were homogenized in 5 ml of CelLytic buffer comprising mild detergent, bicine and 150 mMNaCl (Sigma-Aldrich) and containing a cocktail of protease inhibitors including AEBSF, aprotinin, E-64, EDTA and leupeptin (Pierce) and then centrifuged at 3000 g for 30 min. The supernatant was collected, and the total protein concentration was measured. All samples were adjusted to 1 mg/ml and then stored at -80°C. The samples were thawed on ice directly prior to analysis.

In vivo microdialysis in freely moving mice to sample extracellular α-syn

Transgenic mice overexpressing human α-syn with 50 weeks of age were used in this study. The mice were housed on a 12-hr light/dark cycle with conditions maintained at standard indoor temperature (21 ± 2°C) and humidity (55 ± 5%). Food and tap water were available ad libitum. To enable microdialysis in the hippocampus, mice were anaesthetized with sevoflurane (Abbott

laboratories, Gentofte, Denmark) and an intracerebral guide cannula (brainlink) was stereotaxically implanted into the brain, positioning the microdialysis probe in the hippocampus (co-ordinates of probe tip: 3.1 mm posterior and 2.8 mm lateral from bregma, and 1.0 mm relative dura mater) according to the atlas of Paxinos and Franklin (Paxinos G, Franklin KBJ.The Mouse Brain in Stereotaxic Coordinates, 2nd edition Academic Press, New York, 2001). Anchor screws and acrylic cement were used for the fixation of the guide cannulas. The mice were allowed to recover for 48 h. On the day of the experiment, a microdialysis probe (brainlink, membrane length of 2 mm and a 3 MDamolecular weight cut-off) was inserted through the guide cannula. It was used a microdialysis peristaltic pump (MAB20, Microbiotech A/S) operated in a push-pull mode. The inlet tubing of the microdialysis probe was connected to a peristaltic pump, perfusing the probe with artificial CSF perfusion fluid (in mM: 147 NaCl, 2.7 KCl, 1.2 $CaCl_2$, 0.85 $MgCl_2$) supplemented with 0.2 % bovine serum albumin that was filtered through a 100 kDa MWCO membrane (Millipore, Denmark). The peristaltic pump was also connected to the outlet tubing in order to prevent perfusion fluid loss from the probe, by pulling the fluid through the tubing. The actual flow rate of the pump was determined without having the probe connected. The sample tubes were weighed before and after sampling for a given time period and the flow rate was calculated. The pump was then set to have a constant flow of 0.5 μl/min. A 2h sampling regimen was used throughout the experiment period. To avoid tissue damage, the experimental window was set from 16h after probe implantation. The dialysates were stored at -80 °C until α-syn determination by ELISA.

References

1. Braak H, Del Tredici K, Rüb U, de Vos RA, Jansen Steur EN, Braak E: **Staging of brain pathology related to sporadic Parkinson's disease.** *Neurobiol Aging* 2003, **24:**197-211.
2. Braak H, Alafuzoff I, Arzberger T, Kretzschmar H, Del Tredici K: **Staging of Alzheimer disease-associated neurofibrillary pathology using paraffin sections and immunocytochemistry.** *Acta Neuropathol* 2006, **112:**389-404.
3. Alafuzoff I, Arzberger T, Al-Sarraj S, Bodi I, Bogdanovic N, Braak H, Bugiani O, Del-Tredici K, Ferrer I, Gelpi E, et al: **Staging of neurofibrillary pathology in Alzheimer's disease: a study of the BrainNet Europe Consortium.** *Brain Pathol* 2008, **18:**484-496.
4. Alafuzoff I, Ince PG, Arzberger T, Al-Sarraj S, Bell J, Bodi I, Bogdanovic N, Bugiani O, Ferrer I, Gelpi E, et al: **Staging/typing of Lewy body related alpha-synuclein pathology: a study of the BrainNet Europe Consortium.** *Acta Neuropathol* 2009, **117:**635-652.
5. Neumann M, Müller V, Kretzschmar HA, Haass C, Kahle PJ: **Regional distribution of proteinase K-resistant alpha-synuclein correlates with Lewy body disease stage.** *J Neuropathol Exp Neurol* 2004, **63:**1225-1235.
6. Westerlund M, Ran C, Borgkvist A, Sterky FH, Lindqvist E, Lundströmer K, Pernold K, Brené S, Kallunki P, Fisone G, et al: **Lrrk2 and alpha-synuclein are co-regulated in rodent striatum.** *Mol Cell Neurosci* 2008, **39:**586-591.
